# Prevalence and predictors of premarital sexual intercourse among young women in sub-Saharan Africa

**DOI:** 10.1186/s12978-023-01626-8

**Published:** 2023-06-29

**Authors:** Eugene Budu, Abdul-Aziz Seidu, Ebenezer Kwesi Armah-Ansah, James Boadu Frimpong, Richard Gyan Aboagye, Stephen Kofi Anin, John Elvis Hagan, Bright Opoku Ahinkorah

**Affiliations:** 1 Department of Population and Health, University of Cape Coast, Cape Coast, Ghana; 2 Korle Bu Teaching Hospital, P.O.Box 77, Accra, Ghana; 3 REMS Consult, Takoradi, Ghana; 4 Centre for Gender and Advocacy, Takoradi Technical University, P.O. Box 256, Takoradi, Ghana; 5 College of Public Health, Medical and Veterinary Sciences, James Cook University, Townsville, Australia; 6 Department of Health, Physical Education, and Recreation, University of Cape Coast, Cape Coast, Ghana; 7 Department of Kinesiology, New Mexico State University, Las Cruces, NM USA; 8 Department of Family and Community Health, Fred N. Binka School of Public Health, University of Health and Allied Sciences, Hohoe, Ghana; 9 School of Public Health, Bielefeld University, P.O. Box 100131, 33501 Bielefeld, Germany; 10 Department of Industrial and Health Sciences, Faculty of Applied Sciences, Takoradi Technical University, P.O. Box 256, Takoradi, Ghana; 11 Neurocognition and Action-Biomechanics-Research Group, Faculty of Psychology and Sport Sciences, Bielefeld University, Bielefeld, Germany; 12 School of Public Health, Faculty of Health, University of Technology Sydney, Sydney, Australia

**Keywords:** Premarital sexual intercourse, Sexual and reproductive health, sub-Saharan Africa, Young women

## Abstract

**Introduction:**

Premarital sexual intercourse (PSI) without adequate information and/or appropriate application of the relevant knowledge about sex before marriage, potentially has adverse effects on the sexual and reproductive health outcomes of vulnerable young women in sub-Saharan Africa (SSA). This study sought to examine the prevalence and predictors of PSI among young women aged 15–24 in SSA.

**Methods:**

Nationally representative cross-sectional data from 29 countries in SSA were extracted for the study. A weighted sample size of 87,924 never married young women was used to estimate the prevalence of PSI in each country. A multilevel binary logistic regression modelling approach was used to examine the predictors of PSI at *p* < 0.05.

**Results:**

The prevalence of PSI among young women in SSA was 39.4%. Young women aged 20–24 (aOR = 4.49, 95% CI = 4.34, 4.65) and those who had secondary/higher educational level (aOR = 1.63, 95% CI = 1.54, 1.72) were more likely to engage in PSI compared to those aged 15–19 and those with no formal education. However, young women who belonged to the Islamic religion (aOR = 0.66, 95% CI = 0.56, 0.78); those who were working (aOR = 0.75, 95% CI = 0.73, 0.78); belonged to the richest wealth index (aOR = 0.55, 95% CI = 0.52, 0.58); were not exposed to radio at all (aOR = 0.90, 95% CI = 0.81, 0.99); were not exposed to television at all (aOR = 0.50, 95% CI = 0.46, 0.53); resided in rural areas (aOR = 0.73, 95% CI = 0.70, 0.76); and those who were living in the East African sub-region (aOR = 0.32, 95% CI = 0.29, 0.35) were less likely to engage in PSI compared to those who were traditionalist, unemployed, belonged to the poorest wealth index, exposed to radio frequently, exposed to television frequently, resided in urban areas, and lived in the Southern Africa sub-region, respectively.

**Conclusion:**

Sub-regional variations in the prevalence of PSI exist amidst multiple risk factors among young women in SSA. Concerted efforts are required to empower young women financially, including education on sexual and reproductive health behaviors such as the detrimental effects of sexual experimentation and encouraging abstinence and/or condom use through regular youth-risk communication advocacy.

**Supplementary Information:**

The online version contains supplementary material available at 10.1186/s12978-023-01626-8.

## Plain Language Summary

Having premarital sexual intercourse (PSI) without adequate knowledge and application of the knowledge could have adverse effects on the sexual and reproductive health of vulnerable young women in sub-Saharan Africa (SSA). This study examined the prevalence and predictors of PSI among young women in SSA. Nationally representative cross-sectional data from 29 countries in SSA were used. A sample size of 87,924 never married young women was used to estimate the prevalence of PSI. A multilevel binary logistic regression was used to examine the predictors of PSI. The prevalence of PSI among young women in SSA was high. Young women aged 20–24 and those who had attained secondary/higher educational level were more likely to engage in PSI. However, young women who belonged to the Islamic religion; were working; belonged to the richest wealth index; were not exposed to radio at all; were not exposed to television at all; resided in rural areas; and those who were living in the East African sub-region were less likely to engage in PSI. Sub-regional variations in the prevalence of PSI exist amidst multiple risk factors among young women in SSA. Concerted efforts are required to empower young women financially, including education on sexual and reproductive health behaviors such as the detrimental effects of sexual experimentation and encouraging abstinence and/or condom use through regular youth-risk communication advocacy.

**Supplementary Information:**

The online version contains supplementary material available at 10.1186/s12978-023-01626-8.

## Introduction

The world has seen a significant increase in the total number of young people and it has been estimated that more than three-quarters of the world’s 1.8 billion young people reside in developing regions including sub-Saharan Africa (SSA) [1, 2]. Majority of these young people engage in premarital sexual intercourse (PSI) or sex before they attain their second decade in life [1].

PSI among young women has become a major public health concern as the  world began witnessing a significant increase in reported sexual activities leading to teenage pregnancies and abortions in most developing regions [2–6]. PSI has been explained to encompass penetrative vaginal intercourse that occurs between two individuals before he/she starts a formal marriage life [5, 7–9]. PSI in SSA has been defined as sexual intercourse before attaining age 18 [10, 11]. This is the more vulnerable group of people who are mostly not married and are young [12].

Although PSI is common among young people, it does not translate into contraceptive use [13]. This has had an adverse effect on their sexual and reproductive health outcomes [14, 15]. Young people are involved in PSI without adequate information and knowledge of reproductive health and contraceptives [9, 16–18]. It is essential to note that many life events, health-damaging behaviours, and PSI start at these youthful years [2]. Therefore, the onset of sexual intercourse is a potentially life-changing milestone in the physical and psychological development of women in all settings and the timing and circumstances within which it happens can have either an immediate or long-term consequences on their health and wellbeing [19].

At this stage of development, young women are faced with exploration and risk-taking behaviours [20]. These risky behaviours are associated with pressure to use alcohol, cigarettes, drugs, early  sexual relationships, intentional and unintentional injuries, having multiple sexual partners, violence that could lead to unwanted pregnancy, unsafe abortion, and sexually transmitted infections (STIs) including HIV/AIDS, hasty and unpromising marriage, lesser employment opportunity, unplanned parenthood, and dropping out of school [2, 6, 7, 21].

It has been documented that young women in SSA tend to engage in early sexual debut than their male counterparts and it is one of the major predictors that put young women at a high-risk of HIV/AIDS [11, 22]. This is because majority of the young women are challenged with peer pressure and are shy to discuss sexual matters with their parents explicitly due to culture reasons [1].

Studies have revealed that there are three categories of predictors of PSI among young women in SSA: namely individual, family, and institutional level predictors [12, 22]. The individual-level predictors include age, sex, ethnicity, love affair, and loneliness [12]. The family-level predictors consist of family type, family income, occupation, broken families, and parenting while the institutional level predictors are not only limited to social network, organization, communication (mobile phones, internet, books and magazines, radio and television) but includes policies and laws [12, 23]. Also, studies have indicated that factors such as urban poor settings, high unemployment, unstable wages, low literacy, and inadequate recreational facilities have been linked to young women's engagement in PSI and multiple sexual partners [14, 24, 25].

There is a high prevalence of risky sexual behaviours (e.g., multiple sexual partners, transactional sex, early sexual debut) among young women in SSA [26, 27]. Evidence suggest that by age 15 years, young women in Kenya would have had at least a sexual intercourse in their lifetime while the median age at first sexual intercourse among young women in Ghana and Malawi is 16 years [22].

Studies on sexual practices and behaviours in SSA have mainly focused on adolescents [1]; correlates of early sexual debut [22]; university students [28, 29]; preparatory school students living with and without parents [18]; magnitude and associated factors of premarital sex [2]; in-school youths [16]; parenting style [6]; impact of premarital sex on health [12]; and adolescent students’ attitude towards premarital sex [23].

Since there is a paucity of studies related to the prevalence and predictors of premarital sexual intercourse among young women in SSA, it is essential to understand and identify current trend of this behavioral practice of young women in order to develop appropriate and context-specific interventions in the sub-region. Findings from this study will play an invaluable role in inspiring young women about the risk of premarital sexual practice and inform policymakers as well as for organizations that work in this area.

## Methods

### Data source and study design

Data for the study were extracted from the most recent Demographic and Health Surveys (DHS) of 29 countries in SSA. We pooled the data from the women’s recode files in each of the countries. The DHS is a comparatively nationally representative survey conducted in over 85 low-and-middle-income countries worldwide [30]. DHS employs a descriptive cross-sectional design. Respondents for the survey were recruited using a two-stage cluster sampling method. Detailed sampling technique has been highlighted in the literature [31]. Standardized structured questionnaires were used to collect data from the respondents on health and social indicators including age at first sexual intercourse. We included a total of 87,924 never married young women in our study. Only the women with complete cases of variables of interest were included in the study using listwise deletion. The dataset used is freely available at https://dhsprogram.com/data/available-datasets.cfm. This manuscript was drafted with reference to the Strengthening the Reporting of Observational Studies in Epidemiology (STROBE) statement guidelines [32]. Table 1 provides details of the description of the study sample.

**Table 1:** Description of the study sample

Country	Survey year	Weighted sample (n)	Weighted percentage (%)
1. Angola	2015–16	3974	4.5
2. Burkina Faso	2010	2754	3.1
3. Benin	2017–18	3556	4.0
4. Burundi	2016–17	5071	5.8
5. Congo DR	2013–14	4139	4.7
6. Congo	2011–12	2287	2.6
7. Cote d’Ivoire	2011–12	2294	2.6
8. Cameroon	2018	3650	4.1
9. Ethiopia	2016	3448	3.9
10. Gabon	2012	2284	2.6
11. Ghana	2014	2407	2.7
12. Gambia	2019–20	3095	3.5
13. Guinea	2018	2366	2.7
14. Kenya	2014	3352	3.8
15. Comoros	2012	1426	1.6
16. Liberia	2019–20	2169	2.5
17. Lesotho	2014	359	0.4
18. Mali	2018	1495	1.7
19. Malawi	2015–16	4768	5.4
20. Nigeria	2018	3716	4.2
21. Namibia	2013	2856	3.2
22. Rwanda	2019-2020	3999	4.5
23. Sierra Leone	2019	4080	4.6
24. Senegal	2010–11	3738	4.2
25. Chad	2014–15	2599	3.0
26. Togo	2013–14	2164	2.7
27. Uganda	2016	4165	4.7
28. Zambia	2018	3545	4.0
29. Zimbabwe	2015	2165	2.5
All countries	2010–2020	87,924	100.0

### Variables

#### Outcome variable

The study used PSI as the outcome variable. PSI refers to any sexual relations a person has prior to marriage [33]. Simply put, it is when a person engages in sexual activities before they marry. Restricting the analytical sample to never married young women, this variable was derived using the question on age at first sex which was  “at what age did [NAME] first have sex?” For this study, those who had never had sex were put in the category “No PSI” and this category was coded as “0” while those who had sex at age eight (which was the minimum age at first sex for the study sample in all the countries) and above were put in the category “ever had PSI” and were given the code “1”. Studies that used the DHS dataset employed similar coding [34–36].

#### Explanatory variables

The explanatory variables considered in this study were selected based on their association with PSI from literature [21, 28, 37] and also their availability in the DHS dataset. A total of 10 variables were included in the study. These variables were grouped into individual level and contextual level factors. The individual level factors considered in this study were women’s age, educational level, religious affiliation, occupational status, wealth index, frequency of reading newspaper or magazine, frequency of listening to radio, and frequency of watching television. Place of residence and geographical subregion were the contextual level variables in the study. The categories of each of the variables are shown in Table 2.

**Table 2:** Distribution of premarital sexual intercourse among young women in sub-Saharan Africa across the explanatory variables (n = 87,924)

Variables	Frequency	Percentage	Premarital sexual intercourse	P-value
Age				< 0.001
15–19	62,329	70.9	29.0	
20–24	25,595	29.1	64.6	
Level of education				< 0.001
No formal education	9648	11.0	28.6	
Primary	25,576	29.1	33.0	
Secondary/higher	52,700	59.9	44.4	
Religion				< 0.001
Christianity	61,719	70.2	44.4	
Islam	24,255	27.6	25.8	
Traditional	692	0.8	30.9	
No religion	1258	1.4	56.7	
Employment status				< 0.001
Not working	49,005	55.7	35.7	
Working	38,919	44.3	44.0	
Wealth index				< 0.001
Poorest	10,756	12.2	36.2	
Poorer	13,520	15.4	37.2	
Middle	16,244	18.5	40.0	
Richer	20,020	22.8	41.2	
Richest	27,384	31.1	39.9	
Frequency of reading newspaper or magazine				< 0.001
Not at all	61,699	70.2	37.9	
Less than once a week	13,766	15.7	40.2	
At least once a week	11,539	13.1	44.4	
Almost every day	920	1.0	58.1	
Frequency of listening to radio				< 0.001
Not at all	35,100	39.9	38.2	
Less than once a week	18,424	21.0	38.7	
At least once a week	31,582	35.9	39.2	
Almost every day	2818	3.2	59.0	
Frequency of watching television				< 0.001
Not at all	39,641	45.2	35.7	
Less than once a week	13,054	14.8	39.2	
At least once a week	27,993	31.8	39.4	
Almost every day	7235	8.2	59.6	
Place of residence				< 0.001
Urban	43,434	49.4	45.2	
Rural	44,490	50.6	33.7	
Sub-region				< 0.001
West Africa	31,665	36.0	38.1	
East Africa	37,874	43.1	32.9	
Central Africa	15,169	17.2	53.1	
Southern Africa	3216	3.7	63.6	

### Statistical analyses

Data for the study were analysed using Stata version 16. First, a bar chart was used to show the prevalence of PSI among young women across the 29 countries. Next, the weighted frequencies and percentages for the explanatory variables were presented as indicated in Table 2. Later, we presented the bivariate results on the distribution of PSI among young women across the explanatory variables using the Pearson chi-square test of independence (Table 2). We further conducted a cross-tabulation between age and the other explanatory variables as a sensitivity test to show how age is distributed across the other explanatory variables (Additional file 1). After this, we checked for collinearity multicollinearity among the explanatory variables using the variance inflation factor (VIF) and the results showed no evidence of high collinearity (Maximum VIF = 1.66, Minimum VIF = 1.07 and Mean VIF = 1.31). Finally, a four-model multilevel binary logistic regression (Model O-III) analysis was conducted. Model O was an empty model where no explanatory variable was used. Model I had only the individual level variables. Model II had only the contextual variables while Model III, which was considered as the complete model had both the individual and contextual level variables. The results were presented as adjusted odds ratio (aOR) with their respective 95% confidence interval (CI). All the analyses  were weighted while the survey command (svy) in Stata was used to adjust for the complex sampling structure of the data in the analyses.

### Ethical consideration

In this study, ethical clearance was not sought due to the public availability of the DHS dataset. The datasets were obtained from the Monitoring and Evaluation to Assess and Use Results Demographic and Health Surveys (MEASURE DHS) after registration and approval were given for its usage. All the ethical guidelines concerning the use of secondary datasets in the publication were strictly adhered to. Detailed information about the DHS data usage and ethical standards are available at http://goo.gl/ny8T6X.

## Results

### Prevalence of premarital sexual intercourse among young women in SSA

**Fig. 1:**
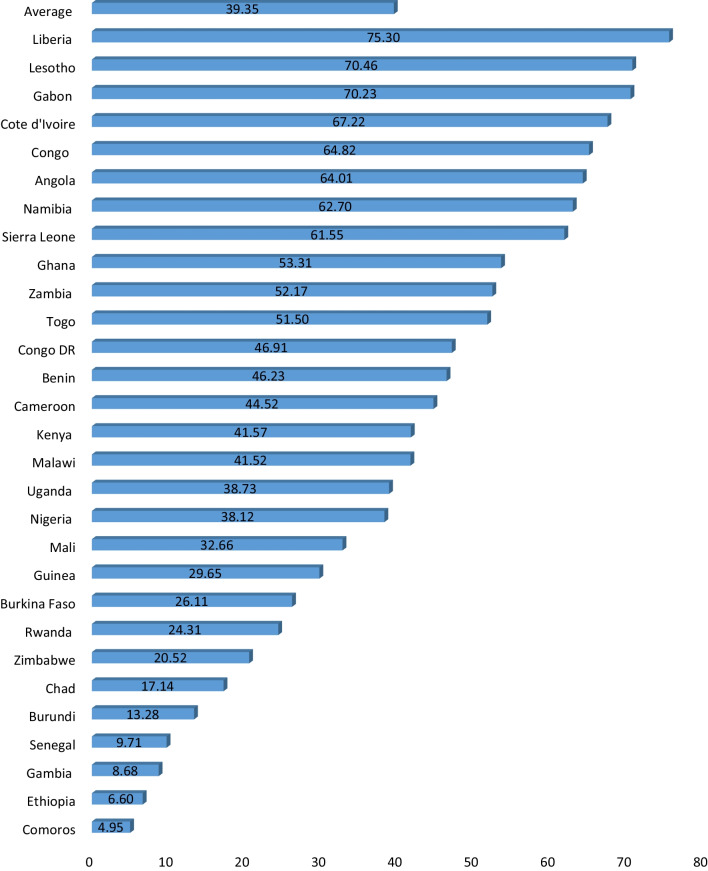
Prevalence (%) of premarital sexual intercourse among young women in sub-Saharan Africa

Figure 1 displays the prevalence of PSI among young women in SSA. The prevalence of PSI among young women in SSA was 39.4%. The country-specific prevalence indicated that Liberia recorded the highest (75.3%) prevalence whereas Comoros recorded the lowest (5.0%) prevalence.

### Association between premarital sexual intercourse and explanatory variables

Table 2 outlines the results on the association between PSI and the explanatory variables. The study found that age, level of education, religion, employment status, wealth index, exposure to newspaper or magazine, exposure to radio, exposure to television, place of residence, and sub-region were all significantly associated with PSI at *p* < 0.001.

### Predictors of premarital sexual intercourse among young women in sub-Saharan Africa

Table 3 presents the results of the predictors of PSI among young women in SSA. The study found that young women aged 20–24 (aOR = 4.49, 95% CI = 4.34, 4.65) and those who had secondary/higher educational level (aOR = 1.63, 95% CI = 1.54, 1.72) had higher odds of engaging in PSI compared to those aged 15–19 and those with no formal education. However, young women who belonged to the Islamic religion (aOR = 0.66, 95% CI = 0.56, 0.78); were working (aOR = 0.75, 95% CI = 0.73, 0.78); belonged to the richest wealth index (aOR = 0.55, 95% CI = 0.52, 0.58); were not exposed to radio at all (aOR = 0.90, 95% CI = 0.81, 0.99); were not exposed to television at all (aOR = 0.50, 95% CI = 0.46, 0.53); resided in rural areas (aOR = 0.73, 95% CI = 0.70, 0.76); and those who were living in the East Africa sub region (aOR = 0.32, 95% CI = 0.29, 0.35) had lower odds of engaging in PSI compared to those who were traditionalist, not working, belonged to the poorest wealth index, exposed to radio almost every day, exposed to television almost every day, resided in urban areas and living in the Southern Africa sub-region, respectively.

**Table 3:** Multilevel binary logistic regression results on the predictors of premarital sexual intercourse among young women in sub-Saharan Africa

Variables	Null model	Model I aOR (95% CI)	Model II aOR (95% CI)	Model III aOR (95% CI)
Age				
15–19		Reference (1.0)		Reference (1.0)
20–24		4.39*** (4.25–4.55)		4.49*** (4.34–4.65)
Level of education				
No formal education		Reference (1.0)		Reference (1.0)
Primary		1.09** (1.03–1.15)		1.34*** (1.26–1.42)
Secondary/higher		1.49*** (1.41–1.58)		1.63*** (1.54–1.72)
Religion				
Christianity		1.49*** (1.26–1.75)		1.74*** (1.47–2.06)
Islam		0.67*** (0.56–0.79)		0.66*** (0.56–0.78)
Traditional		Reference (1.0)		Reference (1.0)
No religion		2.44*** (1.99–2.98)		2.44*** (1.99–3.00)
Employment status				
Not working		0.77*** (0.75–0.80)		0.75*** (0.73–0.78)
Working		Reference (1.0)		Reference (1.0)
Wealth index				
Poorest		Reference (1.0)		Reference (1.0)
Poorer		0.99 (0.94–1.04)		0.99 (0.94–1.05)
Middle		0.96 (0.91–1.01)		0.94* (0.89–0.99)
Richer		0.83*** (0.78–0.87)		0.77*** (0.73–0.82)
Richest		0.59*** (056–0.62)		0.55*** (0.52–0.58)
Frequency of reading newspaper or magazine				
Not at all		1.06 (0.89–1.25)		0.98 (0.83–1.17)
Less than once a week		0.89 (0.75–1.05)		086 (0.72–1.02)
At least once a week		0.98 (0.83–1.17)		0.87 (0.73–1.04)
Almost every day		Reference (1.0)		Reference (1.0)
Frequency of listening to radio				
Not at all		0.82*** (0.74–0.91)		0.90* (0.81–0.99)
Less than once a week		0.73*** (0.66–0.81)		0.80*** (0.72–0.89)
At least once a week		0.69*** (0.62–0.76)		0.79*** (0.71–0.88)
Almost every day		Reference (1.0)		Reference (1.0)
Frequency of watching television				
Not at all		0.42*** (0.39–0.45)		0.50*** (0.46–0.53)
Less than once a week		0.51*** (0.47–0.55)		0.56*** (0.52–0.60)
At least once a week		0.55*** (0.51–0.59)		0.56*** (0.52–0.60)
Almost every day		Reference (1.0)		Reference (1.0)
Place of residence				
Urban			Reference (1.0)	Reference (1.0)
Rural			0.74*** (0.72–0.76)	0.73*** (0.70–0.76)
Sub-region				
West Africa			0.34*** (0.32–0.37)	0.61*** (0.56–0.67)
East Africa			0.28*** (0.26–0.31)	0.32*** (0.29–0.35)
Central Africa			0.65*** (0.60–0.71)	0.78*** (0.71–0.86)
Southern Africa			Reference (1.0)	Reference (1.0)
Random effect results				
PSU variance	0.04 (0.04–0.05)	0.04 (0.04–0.06)	0.05 (0.04–0.06)	0.05 (0.04–0.06)
ICC	0.0126461	0.0132099	0.0141608	0.0137314
LR test	250.07 (X = 0.0000)	214.16 (X = 0.0000)	262.74 (X = 0.0000)	203.97 (X = 0.0000)
Wald chi-square		11,593.86***	2934.43***	13,307.89***
Model fitness				
Log-likelihood	− 58,809.755	− 51,886.136	− 57,288.897	− 50,442.408
AIC	117,623.5	103,816.3	114,589.8	100,936.8
N	87,924	87,924	87,924	87,924
Number of groups	1567	1567	1567	1567

*aOR* adjusted odds ratio, *CI* confidence interval

*p < 0.05, **p < 0.01, ***p < 0.001

## Discussion

The study examined the prevalence and predictors of PSI among young women in SSA. The study found that the pooled prevalence of PSI among young women in SSA was 39.4%. The country-specific prevalence indicated that Liberia recorded the highest (75.3%) prevalence whereas Comoros recorded the lowest (5.0%). The variations in the prevalence of PSI among young women noted in this study could be attributed to the differences in sociocultural practices in these countries. For example, it could be that young women in Comoros normalized premarital sexual behaviours or certain societal norms encouraged sexual experimentation among young women [1], which made them underreport their engagement in PSI. The fear that they would be identified and punished could have also led to the lower prevalence of PSI among young women from Comoros. The highest prevalence of PSI among young women in Liberia could be as a result of the young women’s incessant engagement in transactional sex [38, 39]. Our finding indicates that young women in SSA could be at higher risk of contracting STIs. Therefore, both public and private organizations should intensify their efforts to educate young women about the consequences of engaging in  PSI.

Similar to the findings of other previous studies [1, 16, 20, 40], our study found that the likelihood of PSI among young women heightened with increasing age. A plausible explanation for this finding could be as result of older women engaging in romantic relationships, which increase their likelihood of having PSI [16, 41, 42]. Since younger women aged 15–19 are generally expected to be in school, it is not surprising that they are less likely to have PSI. This finding suggests that sexual and reproductive health education should be targeted at older young women to reduce the occurrence of PSI in SSA.

Akin to the observation of previous study [1], this study found that young women who had secondary/higher educational level had higher odds of engaging in PSI compared to those with no formal education. This finding could be as a result of the influence of peers who convince their colleagues to engage in erotic relationships, which subsequently increases their likelihood of engaging in premarital sex [43, 44]. It could also be that young women who have attained some level of education try to experiment sexual activities that might have been discussed in the schools, increasing their likelihood of having PSI [45, 46]. This finding implies that having higher education is positively associated with PSI, hence, more concerted efforts should be put in educating young women in the school environment about the detrimental effects of sexual experimentation while encouraging abstinence or contraceptive use.

However, young women who belonged to the Islamic religion had lower odds of engaging in PSI relative to those who were traditionalists. This finding is similar to a study [47] which found that being more religious decreased the likelihood of sexual debut among females. Our finding could be attributed to the existence of certain Islamic teachings that frown upon PSI, reducing young women’s likelihood to engage in sexual activities before marriage [47, 48]. Another reason for this finding could be that engaging in more religious activities such as praying five times daily and Quranic recitals protect young Islamic women from engaging in PSI [47].

Young women who were working had lower odds of engaging in PSI compared to those who were not working. Our finding could be that women who are employed are less encouraged to engage in PSI due to the workload which pose as a source of stress on them. Also, women who are unemployed may have the desire to engage in PSI because of curiosity, experimentation and for financial gains to sustain themselves economically [49, 50]. This finding signifies that providing young women with economically sustainable jobs and empowering them financially could help reduce the occurrence of PSI in SSA.

Contrary to the finding of a previous study [51], this study revealed that young women who belonged to the richest wealth index had lower odds of engaging in PSI as against those who belonged to the poorest wealth index. This finding was observed probably because being financially buoyant protects people from engaging in risky sexual behaviors such as streetism, substance use, and transactional sex which increase women’s propensity of having PSI [52].

Despite the well-documented positive influence of mass media exposure (e.g., television and radio) on sexual behavior [26, 53], this study revealed that young women who were not exposed to radio and television at all had lower odds of engaging in PSI compared to those who were exposed to radio and television almost every day. A probable explanation for this unexpected finding could be attributed to the influx of excess sexually explicit telenovelas and advertisement of sexual enhancing drugs that increase the desire of young women to have PSI [54]. For PSI among young women in SSA to reduce significantly, it is important for media outlets to regulate the programs they churn out for public consumption.

Contrary to the finding of previous studies [1, 55], it was found in this study that young women who resided in rural areas had lower odds of engaging in PSI compared to those who resided in urban areas. A plausible explanation for this finding could be linked with the relatively lower standard of living in rural areas, which reduces young women’s likelihood of engaging in transactional sex for financial assistance [56]. Even though this is not clear, this finding calls for further studies to substantiate this probable reason.

Young women who were living in the East Africa sub-region had lower odds of engaging in PSI compared to those who were living in the Southern Africa sub-region. A plausible reason for this finding could be attributed to the variations in the socio-cultural practices among the sub-regions of SSA. For example, it is perceived in some parts of Southern Africa sub-region that having a child increases the chance of marriage, which also increases the rate of PSI among young women [57]. This was evident in this study where Southern African countries such as Angola, Lesotho, Namibia, and Zambia recorded a high prevalence of premarital sex among young women.

## Strengths and limitations

Since the subject was very sensitive, ruling out social desirability bias in relation to the responses was impossible. Additionally, the cross-sectional nature of the study makes it difficult to establish causal inferences among the studied variables. Again, since the study used self-reported questionnaires, it could be that respondents might have underreported or over-reported their experiences. Despite the above-mentioned limitations, the study has certain strengths that need to be mentioned. Hence, findings from the study should be interpreted with caution. First, the study employed a relatively large sample size that is nationally representative and would be appropriate for generalization. Moreover, the use of statistical procedures to generate interesting findings that could be verified is a strength for the study.

## Conclusions

The study revealed that the prevalence of PSI among young women in SSA was high with Liberia and Comoros recording the highest (75.3%) and lowest (5.0%) prevalences, respectively. The study has also identified factors that predict PSI among young women in the studied countries, hence, interventions that seek to alleviate PSI among young women should pay critical attention to these factors. The study recommends that more concerted efforts should be directed at empowering young women financially and educating them about their sexual and reproductive health behaviors such as the detrimental effects of sexual experimentation and also encouraging abstinence and/or contraceptive use.

## Supplementary Information

**Additional file 1: Table S1**. Distribution of age across explanatory variables.

## Data Availability

The dataset used for the study is freely available at https://dhsprogram.com/data/available-datasets.cfm.

## Abbreviations

SSA: Sub-Saharan Africa
PSI: Premarital sexual intercourse
DHS: Demographic and Health Surveys
aOR: Adjusted odds ratio
CI: Confidence interval
STIs: Sexually transmitted illnesses
STROBE: Strengthening the Reporting of Observational Studies in Epidemiology
VIF: Variance inflation factor
