# Supplementary material for: Prevalence and predictors of premarital sexual intercourse among young women in sub-Saharan Africa
Source: Reprod Health. 2023 Jun 29;20:99. doi: 10.1186/s12978-023-01626-8 (PMC10311722; doi:10.1186/s12978-023-01626-8)
Supplement: Supplementary file 1 — Additional file 1: Table S1. Distribution of age across explanatory variables. [file 12978_2023_1626_MOESM1_ESM.docx]

|  | **Age of respondents** | |
| --- | --- | --- |
| **Explanatory variables** | **15-19 years** | **20-24 years** |
| Educational level |  |  |
| No education | 74.63 | 25.37 |
| Primary | 82.36 | 17.64 |
| Secondary/Higher | 64.29 | 35.71 |
| **Religion** |  |  |
| Christianity | 69.42 | 30.58 |
| Islam | 73.86 | 26.14 |
| Traditional | 81.29 | 18.71 |
| No religion | 74.34 | 25.66 |
| **Employment status** |  |  |
| Not working | 76.23 | 23.77 |
| Working | 63.25 | 36.75 |
| **Wealth index** |  |  |
| Poorest | 80.06 | 19.94 |
| Poorer | 77.11 | 22.89 |
| Middle | 74.10 | 25.90 |
| Richer | 69.60 | 30.40 |
| Richest | 62.95 | 37.05 |
| **Exposure to newspaper** |  |  |
| Not at all | 74.11 | 25.89 |
| Less than once a week | 63.67 | 36.33 |
| At least once a week | 62.39 | 37.61 |
| Almost every day | 63.39 | 36.64 |
| **Exposure to radio** |  |  |
| Not at all | 76.30 | 23.70 |
| Less than once a week | 68.98 | 31.02 |
| At least once a week | 66.50 | 33.50 |
| Almost every day | 64.13 | 35.87 |
| **Exposure to television** |  |  |
| Not at all | 76.86 | 23.14 |
| Less than once a week | 69.50 | 30.50 |
| At least once a week | 64.30 | 35.70 |
| Almost every day | 65.49 | 34.51 |
| **Smokes cigarettes** |  |  |
| No | 70.83 | 29.17 |
| Yes | 53.57 | 46.43 |
| **Ever been tested for HIV** |  |  |
| No | 79.78 | 20.22 |
| **Yes** | 52.61 | 47.39 |
| **Place of residence** |  |  |
| Urban | 64.31 | 35.69 |
| Rural | 77.14 | 22.86 |
| **Sub region** |  |  |
| West Africa | 71.19 | 28.81 |
| East Africa | 72.29 | 27.71 |
| Central Africa | 69.48 | 30.52 |
| Southern Africa | 53.85 | 46.15 |
